# Interleukin-6-induced neuroinflammation is exacerbated by subclinical levels of interferon-α

**DOI:** 10.3389/fnins.2025.1586400

**Published:** 2025-06-19

**Authors:** Pattama Songkhunawej, Markus J. Hofer

**Affiliations:** School of Life and Environmental Sciences and Charles Perkins Centre, The University of Sydney, Sydney, NSW, Australia

**Keywords:** interferon, interleukin, inflammation, neurodegeneration, central nervous system, interleukin-6 (IL-6), interferon-α

## Abstract

**Introduction:**

Cerebral cytokinopathies are key examples of dysregulated cytokine responses. While mouse models with targeted production of individual cytokines have been pivotal in establishing a causal link between cytokines and disease— especially in the central nervous system—they often fail to replicate the complex inflammatory environments seen in various neuropathological conditions, such as neuromyelitis optica spectrum disorder, where multiple cytokines are upregulated.

**Methods:**

To address this, we developed a novel mouse model, GFAP-IL6-IFN*^lo^* mice, by combining transgenic mice with astrocyte-targeted production of IL-6 (GFAP-IL6 mice) and IFN-α (GFAP-IFN*^lo^* mice).

**Results:**

Our findings reveal that chronic, low-level production of IFN-α, below the typical disease-inducing threshold, significantly accelerates disease progression in GFAP-IL6-IFN*^lo^* mice compared to GFAP-IL6 mice. The double transgenic mice exhibited progressive ataxia, persistent seizure-like episodes, and reduced survival. Remarkably, the clinical and pathological symptoms remained predominantly IL-6-driven and required the presence of adaptive immune cells.

**Conclusion:**

In summary, we demonstrate that subclinical levels of IFN-α can markedly exacerbate IL-6-mediated neurological disease, suggesting that future studies, should consider the combined effects of IL-6 and IFN-α.

## 1 Introduction

Cytokines play crucial roles in cellular communication within the central nervous system (CNS), during normal homeostasis and in response to infection or injury. As a result, cytokine production is tightly regulated, and dysregulation can increase susceptibility to infections or autoinflammatory diseases. Among the cytokines, interleukin (IL)-6 and the type I interferon (IFN-I) interferon-α (IFN-α) are strongly linked to CNS diseases and their CNS-specific roles have been extensively studied using transgenic mouse models that chronically overexpress IL-6 or IFN-α in the brain, under the control of the astrocyte glial fibrillary acidic protein (GFAP) promoter (GFAP-IL6 and GFAP-IFN mice) (Akwa et al., 1998; Campbell et al., 1993; Campbell et al., 1999).

Concomitant with chronically elevated levels of IL-6 in the CNS, GFAP-IL6 mice exhibit signs of ataxia, neuronal hyperexcitability, and progressive decline in learning function (Campbell et al., 1993; Heyser et al., 1997; Samland et al., 2003). Neuropathological changes in GFAP-IL6 mice include upregulation of acute-phase proteins like complement C3, pro-inflammatory cytokines, astrogliosis and microgliosis, demyelination, and blood–brain barrier (BBB) breakdown and cerebellar volume loss (Barnum et al., 1996; Brett et al., 1995; Campbell et al., 1993; Campbell et al., 2014; Chiang et al., 1994; Gyengesi et al., 2019; Heyser et al., 1997). In myelin oligodendrocyte glycoprotein (MOG)-immunized GFAP-IL6 mice, CNS-specific production of IL-6 shifts inflammation from the typical antigenic site of the spinal cord and markedly enhances disease in the brain (Quintana et al., 2009). This is also observed, albeit at a milder level, in MOG-immunized GFAP-IL6 mice, deficient of systemic IL-6 (GFAP-IL6 × IL-6 knockout mice) (Giralt et al., 2013), highlighting a master regulatory role for CNS-specific IL-6 production.

Conversely, chronic and elevated levels of IFN-α in the CNS of GFAP-IFN*^hi^* mice lead to epileptic seizures, ataxia and increased mortality, accompanied by severe neuropathological changes, including progressive encephalopathy, calcification, and microangiopathy (Akwa et al., 1998; Campbell et al., 1999; Viengkhou et al., 2024a; Viengkhou et al., 2024b). The GFAP-IFN*^hi^* mice have been studied in the context of cerebral interferonopathies, with core clinical and pathological features of Aicardi-Goutières Syndrome (AGS) accurately recapitulated in GFAP-IFN*^hi^* mice (Aicardi and Goutières, 1984; Crow et al., 2015; Lebon et al., 1988). In AGS, the primary source of IFN-α is the CNS, and the microangiopathy and brain disease is strongly associated with CNS-specific IFN-α (Cuadrado et al., 2013; Klok et al., 2015; Lebon et al., 1983; Lodi et al., 2021; van Heteren et al., 2008; Viengkhou et al., 2024b). Importantly, this is independent of peripheral IFN-α blood concentrations in individuals with AGS, indicating a CNS-dominant disease driven by local neurotoxic levels of IFN-α (Viengkhou et al., 2024b). This is further supported by a recent study demonstrating that intracerebroventricular injection of IFN-I receptor-targeted antisense oligonucleotides rescue GFAP-IFN*^hi^* mice from a disease phenotype (Viengkhou et al., 2024a), and highlights an important role for IFN-α produced in the CNS. Notably, mice with lower levels of IFN-α transgene expression (relative to GFAP-IFN*^hi^* mice, but at levels comparable to that observed in the CNS of mice infected with herpes simplex virus or murine hepatitis virus) in the CNS (GFAP-IFN*^lo^*) are relatively unaffected, have a normal life expectancy and show minimal pathological changes in the brain (Akwa et al., 1998; Campbell et al., 1999). This suggests that the otherwise healthy CNS can compensate for chronic, mild increases in IFN-α activity.

While transgenic mice with CNS-targeted production of cytokines have proven valuable in delineating the individual actions of cytokines in the intact CNS, these models do not fully capture the complex cytokine environment seen in many neuropathological conditions. A key example is neuromyelitis optica spectrum disorder (NMOSD), a rare autoimmune disorder of the CNS which primarily affects the optic nerves and the spinal cord. In NMOSD, elevated levels of IL-6 are found in both serum and cerebrospinal fluid (CSF) and strongly correlate with disease severity (Matsushita et al., 2013; Uzawa et al., 2009; Uzawa et al., 2010a; Uzawa et al., 2017). In addition to IL-6, elevated IFN-α levels are observed in the serum of individuals with NMOSD (Asgari et al., 2013), suggesting a role in disease activity. However, undetectable IFN-α levels have been reported in the CNS of individuals with NMOSD (Hofer et al., 2019). Thus, the role of CNS-specific IFN-α in diseases beyond cerebral interferonopathies, and specifically, the pathogenic contribution of IFN-α at chronic, low levels is poorly understood.

To address this, we generated a mouse model that more closely mimics the complex cytokine environment found in human neurological diseases. We achieved this by crossing GFAP-IL6 mice with GFAP-IFN*^lo^* mice, resulting in transgenic mice that chronically overproduce both IL-6 and IFN-α in the CNS (GFAP-IL6-IFN*^lo^* mice). Our findings demonstrate that endogenous production of IFN-α, even at levels below the disease-inducing threshold, is sufficient to significantly exacerbate IL-6-induced neuroinflammation.

## 2 Materials and methods

### 2.1 Animals and ethics

All animal studies were performed in compliance with the NSW Animal Research Act and the 2013 NHMRC “Australian Code of Practice for the care and use of animal for scientific purposes.” Ethics approval was granted by the University of Sydney Animal Care and Ethics Committee (988/1263). GFAP-IFN*^lo^* and GFAP-IL6 mice were bred on a C57BL/6 background while GFAP-IFN*^hi^* mice were on a mixed C57BL/6 × BALB/c background. All mice were maintained under specific-pathogen-free conditions at the animal facility (20–24°C in temperature; 40%–70% humidity; light between 545–1,745 h) at the University of Sydney. GFAP-IFN*^hi^*, GFAP-IFN*^lo^*, and GFAP-IL6 mice were originally developed by I.L. Campbell at the Scripps Research Institute, La Jolla, CA, USA and breeding stock were obtained from there (Akwa et al., 1998; Campbell et al., 1993; Campbell et al., 1999). Genotypes were verified by polymerase chain reaction analysis of tail DNA. Primer sequences: GFAP-IL6 (forward: AGCCAGAGTCCTTCAGAGAGA; reverse: CCGAAAAAACCTCCCACACC) and GFAP-IFN*^hi^*/GFAP-IFN*^lo^* (forward: TGACCCAGCAGATCCTGAAC; reverse: CCGAAAAAACCT CCCACACC). RAG knockout (KO) mice (Mombaerts et al., 1992) were obtained from Animal Resources Centre (ARC; Canning Vale, Australia) and the genotype of RAG KO lines (GFAP-WT × RAG KO, GFAP-IL6 × RAG KO, GFAP-IFN*^lo^* × RAG KO, and GFAP-IL6-IFN*^lo^* × RAG KO) was verified by flow cytometric analysis [CD8a-APC (1:400, 17-0081-81, eBioscience) and CD4-PE (1:400, 12-0041-83, eBioscience)] and by immunohistochemistry of splenocytes to confirm the lack of adaptive immune cells.

Male and female mice were used at random in all experiments and no sex differences were observed. Wildtype (WT) littermates from the breedings were used as controls. Mice were euthanized with isoflurane and brains were collected and bisected at the midline for histological analysis and gene expression or cell signaling analysis.

### 2.2 Motor tests

Motor tests were performed, as previously described (Tung et al., 2014; Viengkhou et al., 2024b), in the Animal Behavioural Facility of The Bosch Institute (now part of the Laboratory Animal Services) at the University of Sydney. For the balance beam, the apparatus consisted of an elevated walking beam (100 cm in length and 1.4 cm in diameter) starting at 52.5 cm above the ground and inclined at an angle toward an enclosed dark box 60 cm above the ground. Following habituation and training, mice were tested for the mean time taken to traverse 60 cm of the beam and the mean number of footslips (when one rear paw slid off the beam) made over five trials. For the rotarod (IITC Life Sciences, Woodland Hills, CA, USA), the apparatus consisted of five separate lanes, each with a horizontally oriented and elevated rod that was 70 mm in width. Mice were habituated on the rods for 1 min prior to testing. The starting speed was set to 4 rpm and linearly accelerated to 40 rpm over 5 min. The mean latency to fall over five trials for each mouse was recorded.

### 2.3 Blood–brain barrier integrity analysis

To assess BBB leakage, Evans blue (EB, 961 Da, E2129, Sigma-Aldrich) and sodium fluorescein (NaFl, 332 Da, 166308, Sigma-Aldrich) were as used per previous studies (Doughty, 2010; Kaya and Ahishali, 2011; Viengkhou et al., 2024b). EB binds strongly to serum albumin and is thus used as a marker to detect leakage of high molecular weight molecules, while NaFl detects leakage of small molecules. Mice were injected intraperitoneally at a volume of 5 ml/kg body weight with 2% (w/v) EB (dissolved in 0.9% (w/v) saline) and left overnight. Mice were subsequently injected intraperitoneally at a volume of 5 ml/kg body weight with 2% (w/v) NaFl (dissolved in 0.9% (w/v) saline). After 30 min, mice were euthanized with isoflurane, rapidly perfused, and brains were collected for visual analysis. To quantify leakage of EB and NaFl, brains were weighed and homogenized in phosphate-buffered saline (PBS). Protein was precipitated from the supernatant homogenate in 25% trichloroacetic acid at 4°C for 30 min. After pelleting at 10,000 g for 10 min at 4°C, the supernatant was prepared for detection of the dyes. For EB, ethanol was added to 70% and for NaFl, sodium hydroxide was added to 1.25 M and standard curves were prepared. Fluorescence was measured using a Tecan Infinite M1000 Pro plate reader (Thermo Fisher Scientific) at an excitation and emission wavelength of 618 ± 5 and 668 ± 5 nm for EB, and of 492 ± 5 and 516 ± 5 nm for NaFl.

### 2.4 Histological analysis

Hemibrains were fixed overnight in neutral buffered 4% paraformaldehyde at 4°C and then processed into paraffin. For histological analysis, 5 μm thick paraffin sections were deparaffinized and rehydrated in graded ethanol. For fresh frozen sections (10 μm), tissues were fixed in acetone:ethanol (3:1) for 5 min at room temperature and rinsed with water. Routine histology [hematoxylin and eosin (H&E) staining] was performed at the Histopathology Core Facility (Department of Pathology, University of Sydney). For immunohistochemistry (IHC), antigen retrieval was performed with 25 mM Tris pH 8, 5 mM EDTA pH 8 and 0.05% SDS (Iba1), or 10 mM citrate pH 6 in 0.05% Tween-20 (CD3) in a vegetable steamer for 40 min. Sections were incubated in 0.3% H_2_O_2_ for 10 min and blocked in 1% goat serum with 0.1% Triton X-100 and 0.05% Tween-20 in PBS for 30 min, then incubated with primary antibodies rabbit anti-Iba1 (1:500, 019-19741, Wako Pure Chemical Industries), rabbit anti-CD3 (1:200, ab16669, Abcam) or rat anti-B220 (1:100, 17-0452-81, eBioscience) overnight at 4°C. Sections were washed in PBS and then incubated with biotinylated anti-rabbit or anti-rat antibodies (1:200, BA-1000 or BA-4001, Vector Laboratories) for 30 min at room temperature, followed by VECTASTAIN Elite ABC HRP Kit (PK-7200, Vector Laboratories) for 30 min. Sections were developed with 3,3′-diaminobenzidine with nickel enhancement (SK-4100, Vector Laboratories), counterstained with Mayer’s hematoxylin and mounted or stained with 2% alizarin red S (ARS; Sigma-Aldrich) before washing in acetone (Chem-Supply) and then coverslipped.

Stained sections were viewed with a DM4000B microscope (Leica Microsystems) and imaged using a SPOT Flex 15.2 64 Mp Shifting Pixel camera and SPOT Advanced 4.5 software (Diagnostic Instruments). Pathology scores were assigned for each cerebellum according to the following criteria: 0 = no observed pathological changes; 1 = higher density or enlarged blood vessels; 2 = moderate degeneration of cerebellum, one large cluster of infiltrates or multiple small clusters; 3 = pronounced degeneration of cerebellum, multiple large clusters of infiltrates. Quantification of CD3 and B220 infiltrates in the cerebellum was performed blinded. For each available mouse, CD3^+^ cells and B220^+^ cells were counted in the cerebella parenchyma over three high power fields (400× magnification). Meningeal infiltrates were not counted, as this was not possible to do accurately for the large meningeal clusters of positive cells in the GFAP-IL6-IFN*^lo^* mice.

### 2.5 Gene expression analysis

Total RNA was isolated from flash frozen cerebella using TRI Reagent (Sigma-Aldrich) according to the manufacturer’s instructions. Concentration and purity of RNA was assessed using a Nanodrop-1000 spectrometer (ThermoFisher) at 260 and 280 nm. For total gene expression analysis, RNase protection assay (RPA) was performed with 10 μg of RNA using specific probes (Asensio and Campbell, 1997; Asensio et al., 1999; Asensio et al., 2001; Hobbs et al., 1993; Maier et al., 2002) and analyzed as described previously (Asensio and Campbell, 1997). For transgene expression analysis, quantitative real-time PCR (qPCR) was performed as described previously using 1 μg of RNA (West et al., 2022a). Primer sequences: transgenic *Il6* (forward: TCACTTTGAGATCTACTCGGCA; reverse: CTGCATTCTAGTTGTGGTTTGTC) (West et al., 2022a) and transgenic *Ifna* (forward: CAATGTGCTGGGAAGACTGA; reverse: CTGCATTCTAGTTGTGGTTTGTC) (West et al., 2022a).

### 2.6 Cell signaling analysis

Immunoblotting was performed as described previously (Wang et al., 2002) using the following antibodies and dilutions: pY705-STAT3 (1:2,000, CST 9131); STAT3 (1:2,000, CST 4904); pY701-STAT1 (1:1,000, CST 9167); pS727-STAT1 (1:2,000, CST 8826); STAT1 (1:1,000, CST 9172); GAPDH (1:100,000, Sigma-Aldrich G8795); Rabbit IgG-peroxidase (1:30,000, Santa Cruz SC2004); Mouse IgG-peroxidase (1:10,000, Sigma-Aldrich A0168).

### 2.7 Statistical analysis

The specific statistical tests used to determine significance are indicated in the figure legends. For all data comparisons, statistical analysis was calculated using Prism version 9 (GraphPad Software) and a *P*-value < 0.05 was considered statistically significant.

## 3 Results

### 3.1 Chronic CNS-targeted production of IL-6 and IFN-α together results in a severe progressive neurological disease in mice

**FIGURE 1:**
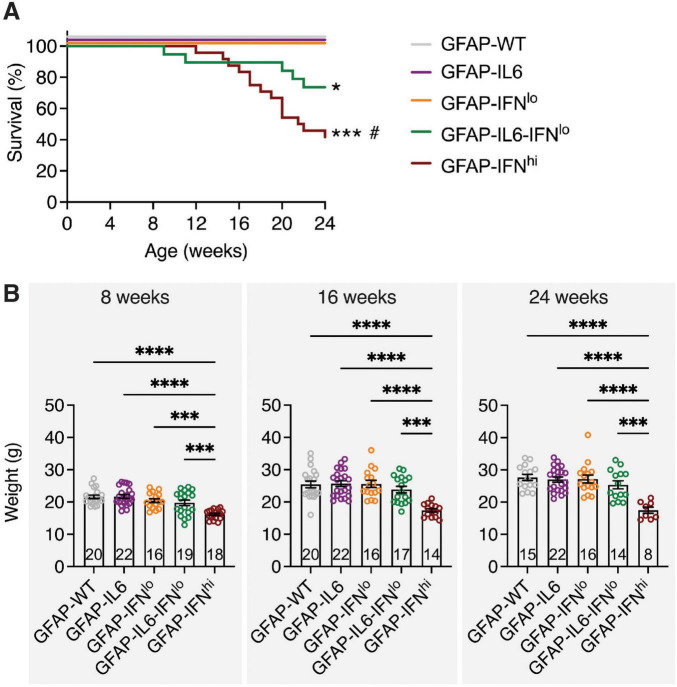
GFAP-IL6-IFN*^lo^* mice have a reduced survival compared with GFAP-WT, GFAP-IL6, and GFAP-IFN*^lo^* mice. **(A)** Survival of mice was recorded over 24 weeks. Significance of survival between genotype groups was calculated using log-rank test. *P < 0.05 and ***P < 0.001 compared with GFAP-WT, GFAP-IL6, or GFAP-IFN*^lo^*; # compared with GFAP-IL6-IFN*^lo^*. **(B)** Weight of mice is shown for 8 (left panel), 16 (middle panel), and 24 (right panel) weeks of age. Graphs show individual mice and mean ± SEM. Significance of weight between genotype groups was calculated using one-way ANOVA with Tukey’s post-test. ***P < 0.001 and ****P < 0.0001. For **(A,B)**, the number of mice is displayed within each bar of panel **(B)**.

To investigate how IL-6-induced neuroinflammation is affected by IFN-α, double transgenic mice with chronic overexpression of both cytokines (GFAP-IL6-IFN*^lo^* mice) were generated and assessed together with WT mice (GFAP-WT mice) and single transgenic mice that chronically overexpress IL-6 (GFAP-IL6 mice) or IFN-α at low levels (GFAP-IFN*^lo^* mice) and at high levels (GFAP-IFN*^hi^* mice) over a 24-week period. Relative to GFAP-WT mice, GFAP-IL6 mice exhibited a mild ataxia by 24 weeks of age, and occasionally, tonic seizures were observed, in line with previous observations (Campbell et al., 1993). In agreement with previous reports (Akwa et al., 1998; Campbell et al., 1999; Hofer et al., 2010; Wang et al., 2002; Wang et al., 2003), GFAP-IFN*^lo^* mice were phenotypically indistinguishable from GFAP-WT mice at these ages. All GFAP-WT, GFAP-IL6, and GFAP-IFN*^lo^* mice survived the 24-week observation period (Figure 1A) and showed no differences in body weight gain (Figure 1B), in contrast to GFAP-IFN*^hi^* mice which had significantly reduced survival, seizures, and were consistently smaller by weight. The double transgenic GFAP-IL6-IFN*^lo^* group also displayed reduced survival, with premature death recorded in approximately 25% of mice by 24 weeks (Figure 1A), although no convulsive seizures or weight loss (Figure 1B) were observed. Of note, progressive motor disease was apparent in GFAP-IL6-IFN*^lo^* mice and past 24 weeks of age, disease in the double transgenic mice was characterized by severe ataxia, and seizure-like tremors and falls (Supplementary Video 1).

**FIGURE 2:**
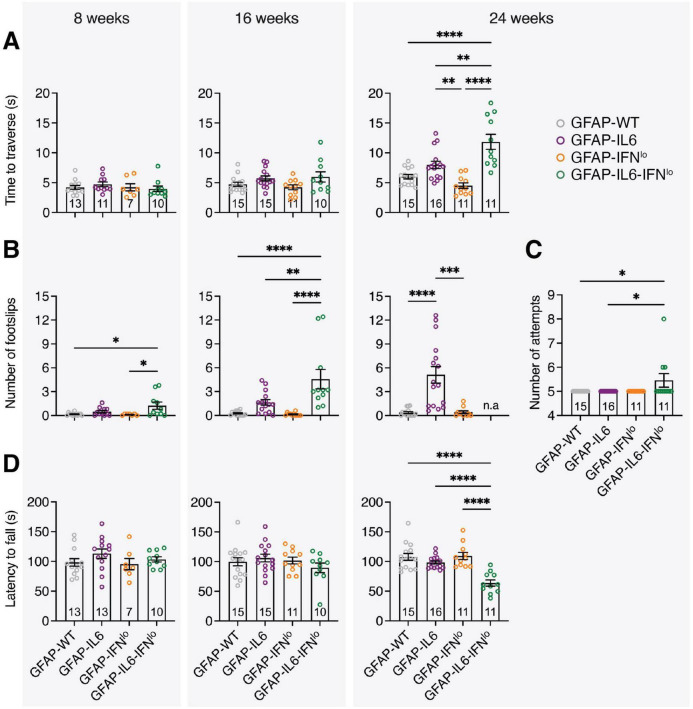
GFAP-IL6-IFN*^lo^* mice develop a more severe and accelerated clinical phenotype compared with GFAP-WT, GFAP-IL6, and GFAP-IFN*^lo^* mice. Motor test performance for mice at 8 (left panel), 16 (middle panel), and 24 (right panel) weeks of age. For the balance beam test, **(A)** the time to traverse the beam, **(B)** the number of footslips when traversing the beam, and **(C)** the number of attempts to complete five trials at 24 weeks of age were recorded. For the rotarod test **(D)**, the latency to fall was measured. For **(A–D)**, graphs show individual mice and mean ± SEM. The number of mice is displayed within each bar. Significance of performance between genotype groups was calculated using one-way ANOVA with Tukey’s post-test. *P < 0.05, **P < 0.01, ***P < 0.001, and ****P < 0.0001.

To quantify the progression of ataxia in these mice, we assessed motor coordination by the balance beam and rotarod tests. In the balance beam test, GFAP-IL6 mice performed similarly to GFAP-WT and GFAP-IFN*^lo^* mice at 8 and 16 weeks of age, traversing the beam in a comparable amount of time and with a low number of footslips (Figures 2A, B). However, at 24 weeks of age, the performance of GFAP-IL6 mice significantly declined, with more footslips compared with either GFAP-WT or GFAP-IFN*^lo^* mice, both of which exhibited no age-dependent changes in motor coordination. Double transgenic mice also displayed an accelerated age-related decline for both performance metrics. By 24 weeks of age, GFAP-IL6-IFN*^lo^* mice performed significantly worse than all other genotype groups, requiring more attempts to complete five trials (Figure 2C). Footslips could no longer be accurately measured for many mice due to the high degree of compensation for balance, where mice curled their tails around the beam and used their rear paws to slide along the beam (Supplementary Video 2). A similar pattern of motor decline was observed in the rotarod test, with a decreased latency to fall recorded only in GFAP-IL6-IFN*^lo^* mice at 24 weeks of age (Figure 2D).

### 3.2 Co-expression of IL-6 and IFN-α exacerbates tissue pathology in the brain of GFAP-IL6-IFN mice

**FIGURE 3:**
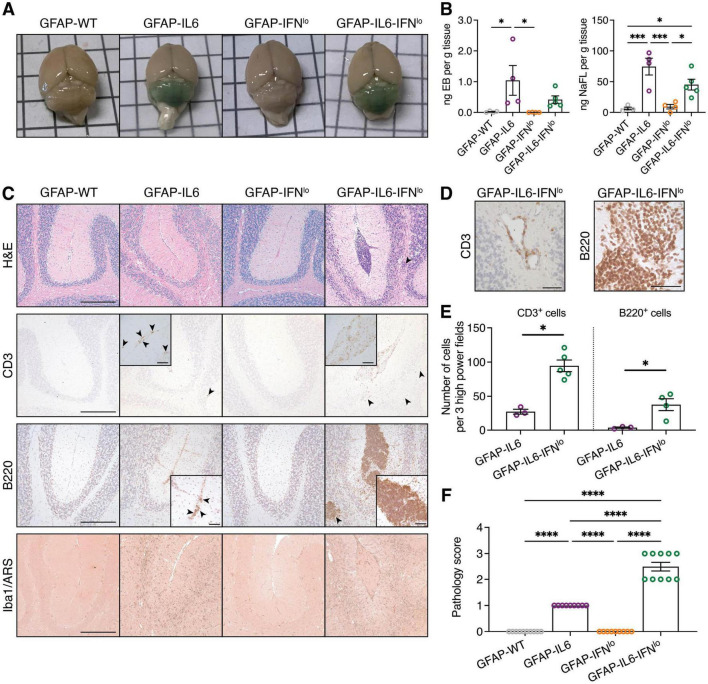
Pronounced pathological features of disease are observed in GFAP-IL6-IFN*^lo^* mice. **(A)** Representative images of the brain of mice following intraperitoneal injection with EB and NaFl. **(B)** Quantification of EB and NaFl extracted from the cerebellum and detected by fluorescence spectroscopy. Graphs show individual mice and mean ± SEM. Representative data of two independent experiments, each with *n* = 4–5, is shown. Significance between genotype groups was calculated by one-way ANOVA with Tukey’s post-test. *P < 0.05 and ***P < 0.001. **(C)** Representative images of H&E staining, CD3, B220, and Iba1/ARS IHC in the cerebellum of mice. Arrowheads indicate areas of parenchymal infiltrates and scale bars represent 250 μm. In inserts, arrowheads indicate meningeal infiltrates for GFAP-IL6 mice and scale bars represent 50 μm. **(D)** Representative images demonstrating perivascular infiltrates in the cerebellum of GFAP-IL6-IFN*^lo^* mice. Scale bars represent 50 μm. For **(A–D)**, *n* = 8–10 mice per genotype group. **(E)** Quantification of T and B cells in the cerebella parenchyma of GFAP-IL6 and GFAP-IFN*^lo^* mice at 24 weeks of age. Graphs show individual mice and mean ± SEM. *n* = 3–5 per genotype group. Significance between genotype groups was calculated by one-tailed Mann–Whitney U test. *P < 0.05. No comparisons were made between immune cell types. **(F)** Pathology scores in the cerebellum of mice at 24 weeks of age. Graphs show individual mice and mean ± SEM. *n* = 8–10 mice per genotype group. Significance of pathology scores between genotype groups was calculated by one-way ANOVA with Tukey’s post-test. ****P < 0.0001.

In GFAP-IL6 mice, a key neuropathological feature is vasculopathy along with BBB disruption, which is most pronounced in the cerebellum, the brain region with the highest transgene expression (Brett et al., 1995; Campbell et al., 1993; Quintana et al., 2009). To test BBB integrity, we assessed accumulation of peripherally injected dyes in the brains of mice at 24 weeks of age. Minimal dye accumulation was observed for GFAP-WT and GFAP-IFN*^lo^* mice. By contrast, the cerebellum of GFAP-IL6 and GFAP-IL6-IFN*^lo^* mice showed green-blue discoloration (Figure 3A) indicative of extravasation of both EB and NaFl into the brain parenchyma and disruption of the BBB allowing molecules of high and low molecular weight, respectively to penetrate into the brain parenchyma. Quantification revealed that accumulation of dyes in the cerebellum was most prominent for GFAP-IL6 mice followed by the GFAP-IL6-IFN*^lo^* mice (Figure 3B).

We next looked at gross pathological changes and immune cell infiltration in the brains of the mice. The cerebellum of 24-week-old GFAP-WT and GFAP-IFN*^lo^* mice showed no overt pathological features (Figure 3C). By contrast, there were moderate pathological changes in the cerebellum of GFAP-IL6 mice, characterized by a loss of Purkinje cells and neurons in the granule cell layer. We also observed abnormal blood vessels as well as few infiltrating CD3^+^ T cells and B220^+^ B cells into the molecular layer and white matter tracts of the cerebellum. These pathological changes were substantially more pronounced in GFAP-IL6-IFN*^lo^* mice, resulting in focal loss of tissue architecture in the cerebellum. This was accompanied by single or multiple large clusters of meningeal infiltrates, comprising primarily of B220^+^ B cells and some CD3^+^ T cells and Iba1^+^ monocytes/macrophages, with occasional perivascular infiltrates (Figure 3D). Quantification of CD3^+^ T cells and B220^+^ B cells in the cerebellar parenchyma also revealed significantly increased infiltration in GFAP-IL6-IFN*^lo^* mice compared with GFAP-IL6 mice (Figure 3E).

Further, previous studies using GFAP-IL6 mice demonstrate a significant microgliosis in the cerebellum (Gyengesi et al., 2019; West et al., 2022a; West et al., 2022b). Compared with GFAP-WT and GFAP-IFN*^lo^* mice, Iba1 staining revealed strong microgliosis, particularly in the white matter of GFAP-IL6 and GFAP-IL6-IFN*^lo^* cerebella, although differences in staining intensity was not apparent between these two genotypes. Notably, calcification, a key hallmark feature of IFN-driven disease in older GFAP-IFN*^lo^* mice and in the higher IFN-α expressing GFAP-IFN*^hi^* mice (Akwa et al., 1998; Campbell et al., 1999; Viengkhou et al., 2024a; Viengkhou et al., 2024b), was not observed by ARS staining in mice. Scoring of the severity of pathological changes confirmed that gross histopathological changes in the cerebellum are exaggerated and more prominent in GFAP-IL6-IFN*^lo^* mice compared with GFAP-IL6, GFAP-IFN*^lo^*, and GFAP-WT mice (Figure 3F). Taken together, the production of IL-6 and IFN-α together in the brain resulted in a tissue pathology similar to that observed in GFAP-IL6 mice, albeit considerably more severe and accelerated.

### 3.3 An exaggerated IFN-induced pro-inflammatory signature is observed in the cerebellum of GFAP-IL6-IFN mice

Behavioral and histopathological assessment of GFAP-IL6-IFN*^lo^* mice had revealed a progressive disease that predominantly reflected the actions of IL-6 in the brain. To further evaluate the impact of chronic production of IL-6 and IFN-α together in GFAP-IL6-IFN*^lo^* mice, the degree of activation of the respective signaling pathways was examined.

**FIGURE 4:**
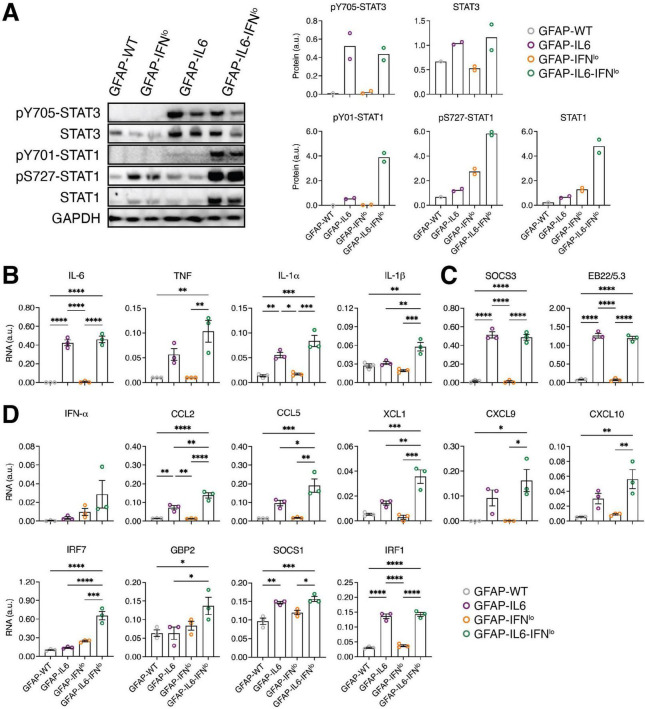
An exaggerated IFN-signature is observed in GFAP-IL6-IFN*^lo^* mice. **(A)** Representative immunoblot data of three independent experiments, each with *n* = 1–2 mice per genotype group, is shown. (Left panel) Immunoblots for STAT3 and STAT1 activation in the cerebellum of mice at 24 weeks of age. (Right panel) Densitometric quantifications of immunoblots, normalized to GAPDH. Graphs show individual mice and mean. **(B–D)** Gene expression in the cerebellum of mice at 24 weeks of age for prototypic **(B)** pro-inflammatory genes, **(C)** IL-6-regulated genes, and **(D)** IFN-regulated genes, normalized to L32. For **(B–D)**, graphs show individual mice and mean ± SEM. *n* = 3 mice per genotype group. Significance between genotype groups was calculated by one-way ANOVA with Tukey’s post-test. *P < 0.05, **P < 0.01, ***P < 0.001, and ****P < 0.0001.

Tyrosine 705 phosphorylation and tyrosine 701 phosphorylation are critical for signal transducers and activators of transcription (STAT) 3 and 1 activation, respectively. Immunoblots revealed no detectable tyrosine phosphorylation of STAT3 (pY705-STAT3) in the cerebellum of GFAP-WT or GFAP-IFN*^lo^* mice at 24 weeks of age (Figure 4A). By contrast, pY705-STAT3 levels were elevated to comparable degrees in both GFAP-IL6 and GFAP-IL6-IFN*^lo^* mice. Similarly, tyrosine phosphorylation of STAT1 (pY701-STAT1) was not detectable by immunoblot in the cerebellum of GFAP-WT or GFAP-IFN*^lo^* mice but was weakly detectable in the cerebellum of GFAP-IL6 mice and elevated in GFAP-IL6-IFN*^lo^* mice. In addition, serine 727 phosphorylation of STAT1 (pS727-STAT1), which is thought to increase STAT1 transcriptional activity (Decker and Kovarik, 2000), was low in GFAP-WT and GFAP-IL6 mice. The level of pS727-STAT1 appeared notably higher in GFAP-IFN*^lo^* mice and was further increased in GFAP-IL6-IFN*^lo^* mice.

Previous findings have shown that STAT1 is activated in the brain of both GFAP-IL6 and GFAP-IFN*^lo^* mice (Hofer et al., 2010; Sanz et al., 2008). Further, the preceding observations suggest that STAT1 activation may contribute to the evolution of the inflammatory response in the brain, mediated by astrocyte-targeted production of IL-6 and IFN-α together. Thus, the expression of pro-inflammatory genes (Figure 4B and Supplementary Figure 1), IL-6-regulated genes (Figure 4C and Supplementary Figure 1) and IFN-regulated genes (Figure 4D and Supplementary Figure 1) in the cerebellum of 24 week old mice was examined. Analysis of pro-inflammatory genes and IL-6-regulated genes showed significantly increased expression of *Il6*, *Il1a*, *Socs3*, and *Eb22/5.3* in GFAP-IL6 mice compared with both GFAP-IFN*^lo^* and GFAP-WT mice (Figures 4B, C). With the exception of *Tnf* and *Il1b* where expression was elevated (compared with GFAP-IFN*^lo^* and GFAP-WT mice for *Tnf* and compared with all three genotype groups for *Il1b*), the observed gene expression profile was comparable in GFAP-IL6-IFN*^lo^* mice, suggesting that IFN-α has a minor effect on the key pro-inflammatory and IL-6-induced gene signatures of the brain.

Analysis of prototypic IFN-regulated genes revealed no significant differences in expression between GFAP-WT and GFAP-IFN*^lo^* mice (Figure 4D). By contrast, *Ccl2*, *Socs1*, and *Irf1* were upregulated in expression in GFAP-IL6 mice compared to GFAP-WT mice, indicating IL-6-induced STAT1 activation. Further, there was a significant increase in the expression of *Ccl2*, *Ccl5*, *Xcl1*, and *Irf7* in GFAP-IL6-IFN*^lo^* mice compared with all three genotype groups. Similarly, gene expression for *Gbp2* was upregulated compared with GFAP-WT and GFAP-IL6 mice while *Cxcl9* and *Cxcl10* were upregulated in expression compared with GFAP-WT and GFAP-IFN*^lo^* mice.

### 3.4 Adaptive immune cells contribute to the neurodegenerative disease observed in double transgenic mice

**FIGURE 5:**
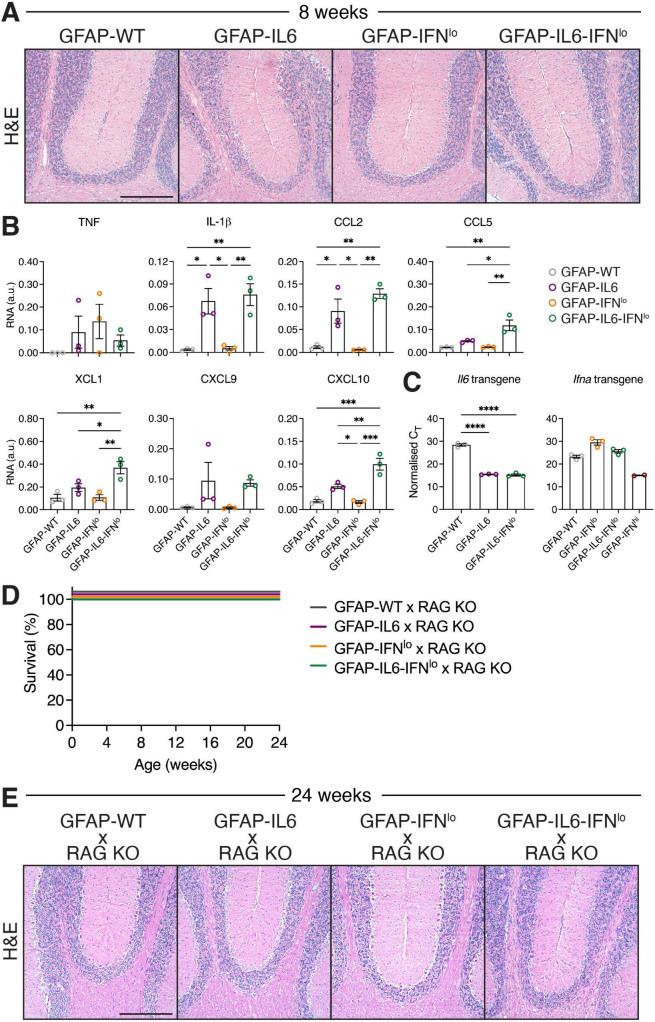
The severe disease in GFAP-IL6-IFN*^lo^* mice is dependent on the recruitment of adaptive immune cells into the CNS. **(A)** Representative images of H&E staining of the cerebellum of mice at 8 weeks of age. Scale bars represent 250 μm. *n* = 5–8 mice per genotype group. **(B)** Gene expression in the cerebellum of mice at 8 weeks of age, normalized to L32. **(C)** Transgene expression analysis of IL-6 and IFN-α, normalized to the C_T_ of the housekeeping gene 18S rRNA. For **(B,C)**, graphs show individual mice and mean ± SEM. *n* = 3 mice per genotype group. Significance was calculated by one-way ANOVA with Tukey’s post-test. *P < 0.05, **P < 0.01, ***P < 0.001, and ****P < 0.0001. **(D)** Survival of mice was recorded over 24 weeks. Significance of survival between genotype groups was calculated using log-rank test. **(E)** Representative images of H&E staining in the cerebellum of mice at 24 weeks of age. Scale bars represent 250 μm. For **(D,E)**, *n* = 5–8 mice per genotype group.

Although cerebellar BBB integrity was compromised in both GFAP-IL6 and GFAP-IL6-IFN*^lo^* lines, distinctly large populations of adaptive immune cell infiltrates was observed only in the brain of GFAP-IL6-IFN*^lo^* mice, and our gene expression analysis revealed significant upregulation of several chemokines and some pro-inflammatory cytokines. To further investigate if increased B and T cell infiltration may be linked to elevated chemokine levels, we determined the level of gene expression for those significantly upregulated at 24 weeks of age, in the brain of mice at 8 weeks. At this age, clinical phenotype is comparable between mice (Figures 1, 2) and few pathological changes are observed in the brain of GFAP-IL6-IFN*^lo^* mice (Figure 5A). In the cerebellum, significant upregulation in expression of *Il1b, Ccl2*, and *Ccl5* was observed but was comparable between both GFAP-IL6 and GFAP-IL6-IFN*^lo^* mice (Figure 5B and Supplementary Figure 2). By contrast, gene expression of *Ccl5, Xcl1*, and *Cxcl10* was significantly upregulated in GFAP-IL6-IFN*^lo^* mice compared with single transgenic GFAP-IL6 and GFAP-IFN*^lo^* mice, and GFAP-WT mice, indicating an early inflammatory response to IL-6 and IFN-α together in the brain. To further elucidate if this could be attributed to changes in transgene production in the CNS, transgene expression was measured at 8 weeks of age. Expression of the *Il6* transgene was comparable between GFAP-IL6-IFN*^lo^* and GFAP-IL6 mice while expression of the *Ifna* transgene was not detectable in either GFAP-IL6-IFN*^lo^* or GFAP-IFN*^lo^* mice by this method (Figure 5C).

Next, to briefly examine the extent to which adaptive cells are involved in disease, we crossed GFAP-IL6-IFN*^lo^* mice with RAG KO mice to generate mice with CNS-restricted production of both IL-6 and IFN-α and a lack of mature T and B cells. In the absence of RAG, GFAP-WT, GFAP-IL6, and GFAP-IFN*^lo^* mice survived past 24 weeks of age (Figure 5D). Similarly, all GFAP-IL6-IFN*^lo^* × RAG KO mice survived by 24 weeks of age but showed mild signs of ataxia which was comparable to GFAP-IL6 mice. Examination of H&E-stained sections of the cerebellum of GFAP-WT × RAG KO and GFAP-IFN*^lo^* × RAG KO mice revealed no overt pathology (Figure 5E). Compared with these mice, vacuolated white matter and moderate degeneration of the granule cell layer was observed in the cerebellum of GFAP-IL6 mice. This was similarly observed in GFAP-IL6-IFN*^lo^* × RAG KO mice. As expected, the large clusters of meningeal infiltrates observed in GFAP-IL6-IFN*^lo^* mice were absent in GFAP-IL6-IFN*^lo^* × RAG KO mice. These findings show that the adaptive immune response plays an important role in mediating the adverse actions of IL-6 and IFN-α together, and suggest that IFN-α accelerates infiltration of B and T cells in the CNS.

## 4 Discussion

Using existing transgenic mice with CNS-restricted production of IL-6 and IFN-α, we created a novel mouse model with combined overproduction of both cytokines to more accurately replicate the complex environment seen in neuroinflammation. Our findings reveal that while subclinical levels of IFN-α alone in the CNS do not cause disease, they are crucial for the development of severe disease in cases where the initial inflammation induced by IL-6 is relatively mild.

### 4.1 Pronounced IL-6 driven disease is exacerbated by IFN-α

Astrocyte-targeted production of IL-6 in GFAP-IL6 mice creates a mild pro-inflammatory environment that progresses gradually with age, though it has minimal impact on survival. This disease is exacerbated in the presence of low levels of IFN-α in GFAP-IL6-IFN*^lo^* mice. Importantly, this exacerbated disease retained key features of the mild disease in GFAP-IL6 mice, such as progressive motor decline, infiltration of B and T cells and cerebellar BBB leakage (Brett et al., 1995; Campbell et al., 1993; Campbell et al., 2014; Chiang et al., 1994; Gyengesi et al., 2019; Heyser et al., 1997). Based on these findings, we propose that elevated levels of IL-6 alone in the CNS may not be sufficient to induce a severe disease, and that other inflammatory pathways including the IFN-α signaling pathway are required to promote the pathogenic actions of IL-6.

We observed increased activation of STAT1 and elevated expression of several IFN-regulated genes in GFAP-IL6-IFN*^lo^* mice. Despite this, the disease in the double transgenic mice lacked key features of IFN-I-instigated neuroinflammation that are seen in GFAP-IFN*^hi^* mice (Akwa et al., 1998; Campbell et al., 1999; Viengkhou et al., 2024b) or individuals with AGS (La Piana et al., 2016; Viengkhou et al., 2024a; Viengkhou et al., 2024b) including reduced growth, convulsive seizures and calcifications. Transgenic expression of *Il6* and presumably *Ifna* remained comparable and endogenous expression of *Il6* or *Ifna* did not differ between GFAP-IL6, GFAP-IFN*^lo^*, and GFAP-IL6-IFN*^lo^* mice, demonstrating that IFN-α does not induce IL-6 overproduction and vice versa. Together, this further points to an IL-6-driven disease enhanced by IFN-α rather than an IFN-α-mediated disease.

The absence of overt disease or pathological changes in low IFN-α expressing GFAP-IFN*^lo^* mice is in sharp contrast to the GFAP-IFN*^hi^* mice (Akwa et al., 1998; Campbell et al., 1999; Viengkhou et al., 2024b) and indicates a threshold level for IFN-α-induced neurotoxicity. The beneficial effects of chronically low levels of raised IFN-α are aligned with its protective role in viral infections. Previous studies have shown that GFAP-IFN*^lo^* mice are resistant to infections by neurotropic lymphocytic choriomeningitis virus (Akwa et al., 1998) and herpes simplex virus (Carr et al., 1998). Further, basal expression of IFN-α is observed in the brain and is essential for development and health (Ejlerskov et al., 2015; Hosseini et al., 2020; Ivashkiv and Donlin, 2014). Basal IFN-I signaling also enhances cellular responses to IL-6 (Mitani et al., 2001; Murray et al., 2015) and IFN-I may support IL-6-mediated B cell survival and differentiation (Jego et al., 2003). Despite a range of beneficial effects, our findings show that IFN-α, below a disease-inducing threshold, can become pathogenic and sufficient to significantly aggravate disease, highlighting its dual role in the CNS.

Signaling specificity in the GFAP transgenic mouse model has been demonstrated in a recent study where microglia in GFAP-IL6 and GFAP-IFN*^hi^* mice exhibited distinct responses despite the pleiotropic effects of IL-6 and IFN-α, as well as the secondary effects which are induced by chronic neuroinflammation (West et al., 2019). Given this signaling specificity, it is intriguing that the GFAP-IL6-IFN*^lo^* mice exhibit such pronounced IL-6-induced disease, especially since GFAP-IFN*^lo^* mice show minimal, if not any, clinical or pathological changes. This raises the hypothesis that blocking IL-6 trans-signaling in the brain of GFAP-IL6-IFN*^lo^* mice might alleviate the detrimental effects of IL-6, consistent with previous findings in the GFAP-IL6 mice where co-production of soluble gp130, an IL-6 trans-signaling inhibitor, reduced disease severity (Campbell et al., 2014). Furthermore, as CNS-resident cells, such as microglia, are responsive to both IL-6 and IFN-α (West et al., 2019), it is tempting to speculate that simultaneous stimulation by both cytokines enhances STAT1 activation, promoting the formation of STAT1 homodimers and possibly STAT1:STAT3 heterodimers. However, how CNS-resident cells as well as infiltrating adaptive immune cells respond to the combined cytokine signaling requires further investigation.

### 4.2 A dependence on immune cell infiltration into the CNS

The absence of mature B and T cells, which prominently infiltrate the brains of GFAP-IL6-IFN*^lo^* mice, rescued double transgenic mice from severe disease. The dependence of the severe disease in the GFAP-IL6-IFN*^lo^* mice on adaptive immune cells could indicate the presence of an autoimmune rather than autoinflammatory pathology, although the presence of autoantibodies or autoreactive T cells remains to be shown. In this context, leakage of small amounts of IL-6 into the peripheral compartment of GFAP-IL6 has been previously proposed (Giralt et al., 2013), and this leakage—potentially combined with IFN-α in GFAP-IL6-IFN*^lo^* mice—might trigger a peripheral adaptive immune response. While the mechanisms initiating such an autoimmune/autoinflammatory response remain uncertain, our findings demonstrate that it requires co-stimulation such as by mildly enhanced IFN-α signaling.

Previous studies using GFAP-IL6 and GFAP-IFN*^lo^* mice have demonstrated that STAT1 is critical for modulating the inflammatory effects of IFN-α but not IL-6 in the brain (Sanz et al., 2008; Wang et al., 2002). GFAP-IL6 mice deficient of STAT1 are phenotypically indistinguishable from GFAP-IL6 mice, despite moderately decreased levels of several pro-inflammatory cytokines (Sanz et al., 2008). By contrast, STAT1 deficiency in GFAP-IFN*^lo^* markedly exacerbates the pathogenic effects of IFN-α, resulting in increased levels of pro-inflammatory cytokines and chemokines, as well as activation of STAT3 but not other factors downstream of IL-6 and IFN-α (Wang et al., 2002). Prioritizing these markers and the Janus kinase (JAK)/STAT pathway in our examination, we found that prior to development of a clinical disease phenotype and a pronounced pathology, the inflammatory profile of GFAP-IL6 and GFAP-IL6-IFN*^lo^* mice could be distinguished by increased levels of only a few chemokines (XCL1, CCL5, and CXCL10), indicating an early and localized response of the CNS.

Interestingly, increased production of CCL5 and CXCL10 is also observed from CNS-resident cells following viral infections of the CNS (Asensio et al., 1999; Wang and Campbell, 2005), with CXCL10 linked to effector T cell trafficking to the CNS (Dufour et al., 2002). This together links the JAK/STAT pathway to chemotactic functions and supports the notion that CNS-specific IFN-α accelerates infiltration of immune cells. This further raises the question of whether such chemokines could be therapeutic targets. However, it should be noted that due to the presence of multiple signaling pathways downstream of IL-6 and IFN-α, it is difficult to ascribe this to a direct effect of the two cytokines in the CNS. In any event, our findings demonstrate that chronic IL-6 and IFN-α signaling in the intact CNS is sufficient to initiate and maintain immune cell recruitment and exacerbate disease.

### 4.3 Accumulating evidence of a role for the IFN signaling pathway in neuroinflammation

While the GFAP-IL6 mice (as well as the GFAP-IL6-IFN*^lo^* mice) are not an animal model of NMOSD (Duan and Verkman, 2020), as characteristic features of NMOSD including optic neuritis and AQP4-IgG-mediated astrogliosis have not been reported, our findings align with recent evidence that IFN-I can exacerbate IL-6-driven inflammation, such as that observed in NMOSD. In individuals with NMOSD, elevated IL-6 levels are associated with disease severity (Matsushita et al., 2013; Uzawa et al., 2009; Uzawa et al., 2010a; Uzawa et al., 2017) and tissue pathology, including BBB leakage and B cell infiltration, largely reflecting the actions of IL-6 (Fujihara et al., 2020). Neutralizing the IL-6R has been shown to effectively alleviate symptoms (Araki et al., 2013; Araki et al., 2014; Ayzenberg et al., 2013; Ringelstein et al., 2015). However, there is also a correlation between IFN-α levels and disease progression (Agasing et al., 2020; Asgari et al., 2013; Feng et al., 2012).

A subset of individuals with NMOSD who exhibit both elevated levels of IL-6 and a heightened IFN-I signature, also show increased levels of cytokines related to the pathogenic T helper 17 cell pathway (Agasing et al., 2020). This IFN-I signature in NMOSD may also reflect a type II IFN (IFN-II) imbalance (Agasing et al., 2020) and is relevant given that IFN-β treatment exacerbates disease in IFN-II-deficient models of NMOSD (Arellano et al., 2024). A fundamental role for IFN-α is further underscored by the observation that IFN-β therapy is ineffective in NMOSD and may even increase relapse rates (Jarernsook et al., 2013; Kim et al., 2012; Palace et al., 2010; Uzawa et al., 2010b), as well as by several reports of NMOSD development following IFN-α therapy (Gao et al., 2019; Rao et al., 2022; Williams et al., 2020; Yamasaki et al., 2012).

### 4.4 Limitations of the study and future directions

Following prolonged IFN-α treatment, glial cells respond with a transient increase in pY701-STAT1 and a prolonged increase in pS727-STAT1 (Li et al., 2017). Similarly, in GFAP-IFN*^hi^* and GFAP-IFN*^lo^* mice, chronic IFN-α signaling in the brain induces a shift in phosphorylation of STAT1, away from tyrosine 701, to serine 727 (Hofer et al., 2010; Viengkhou et al., 2024b). While serine 727 phosphorylation of STAT was detected in GFAP-IFN*^lo^* and GFAP-IL6-IFN*^lo^* mice in this study, indicating chronic IFN-α signaling, the absence of detectable IFN-α transgene expression presents some limitations in interpretation. Previous studies have indicated that leakage of IL-6 and IFN-α to the periphery is plausible in the GFAP-IL6 and GFAP-IFN*^hi^* mice, respectively (Giralt et al., 2013; Viengkhou et al., 2024b). As our findings indicate a significant role for low levels of IFN-α, we cannot exclude that minor leakage of IFN-α, together with IL-6, to the peripheral compartment is sufficient to induce an autoimmune response. Further, it remains plausible that subtle undetectable differences in IFN-α transgene expression contribute to the aggravated disease and the exaggerated IFN signature observed in GFAP-IL6-IFN*^lo^* mice. Nonetheless, our findings indicate that the CNS is highly responsive to IFN-α at chronic low levels.

Interestingly, serine 727 phosphorylated STAT1 was notably increased in the brains of GFAP-IL6-IFN*^lo^* mice and to a lesser extent in GFAP-IFN*^lo^* mice compared with the other genotypes. Given that serine 727 phosphorylation enhances STAT1 transcriptional activity in response to cytokines (Kovarik et al., 2001; Nguyen et al., 2001; Nguyen et al., 2003; Wen et al., 1995; Zhang et al., 1998; Zhu et al., 1997), and that our findings suggest IFN-α is important for exacerbating IL-6-induced inflammation, it is possible that pS727-STAT1 plays a key role in mediating the neuroinflammatory disease in GFAP-IL6-IFN*^lo^* mice. Additionally, the transient levels of pY701-STAT1 suggest that the CNS inhibits prolonged or excessive IFN-I responses with no effect on pS727-STAT1, possibly to protect it from IFN-I-induced neurotoxicity without compromising the antiviral/immune response. However, further studies are needed to better understand the role of pS727-STAT1 in the CNS response to cytokines, and in particular, in chronic IFN-I neurotoxicity.

Previous studies have shown that serine 727 phosphorylation of STAT1 correlates with IFN-I activity in the context of inflammation. In mononuclear cells from individuals with clinically stable NMOSD, IFN-β-induces a significant increase in pS727-STAT1, but not pY701-STAT1, compared with healthy controls. This is associated with elevated levels of MxA, a prototypical IFN-I target (Feng et al., 2012). By contrast, a pS727-STAT1-resistant state in individuals with relapsing-remitting multiple sclerosis correlates with suboptimal responses to IFN-β therapy (Feng et al., 2002). Similarly, in a study on viral neuropathogenesis, human immunodeficiency virus type I induces serine 727 phosphorylation of STAT1 and STAT3 but not tyrosine phosphorylation, which is linked to IL-6 expression, BBB damage, monocyte migration, and neurodegeneration (Chaudhuri et al., 2008). We speculate that serine 727 phosphorylation of STAT1 contributes to and could serve as an accurate marker of (neuro)inflammation. Ongoing work by us, leveraging the transgenic mouse models from the present study and STAT1 knock-in mice (Varinou et al., 2003), seek to uncover this.

## 5 Conclusion

In summary, our study reveals for the first time that chronic overproduction of IFN-α, below the disease-inducing threshold, significantly exacerbates the neuropathological effects of IL-6, highlighting the exquisite responsiveness of the CNS. The localized overproduction of IL-6 together with IFN-α at low levels in the brain is sufficient to induce significant adaptive immune cell infiltration which markedly enhances disease. Our novel animal model can serve as a clinically relevant platform to further study the complex interplay between IL-6 and IFN-I signaling in the living CNS, including the role of STAT1 as a point of convergence between the two pathways. Furthermore, our study underscores the clinical relevance of IFN-α activity and positions the IFN-α signaling pathway as a potential therapeutic target for IL-6-driven diseases. It also highlights the importance of assessing the degree of IL-6-induced inflammation in individuals before prescribing IFN-α therapies.

## Data Availability

The original contributions presented in this study are included in this article/Supplementary material, further inquiries can be directed to the corresponding author.
