# Supplementary figures and images for: Interleukin-6-induced neuroinflammation is exacerbated by subclinical levels of interferon-α
Source: Front Neurosci. 2025 Jun 19;19:1586400. doi: 10.3389/fnins.2025.1586400 (PMC12223566; doi:10.3389/fnins.2025.1586400)

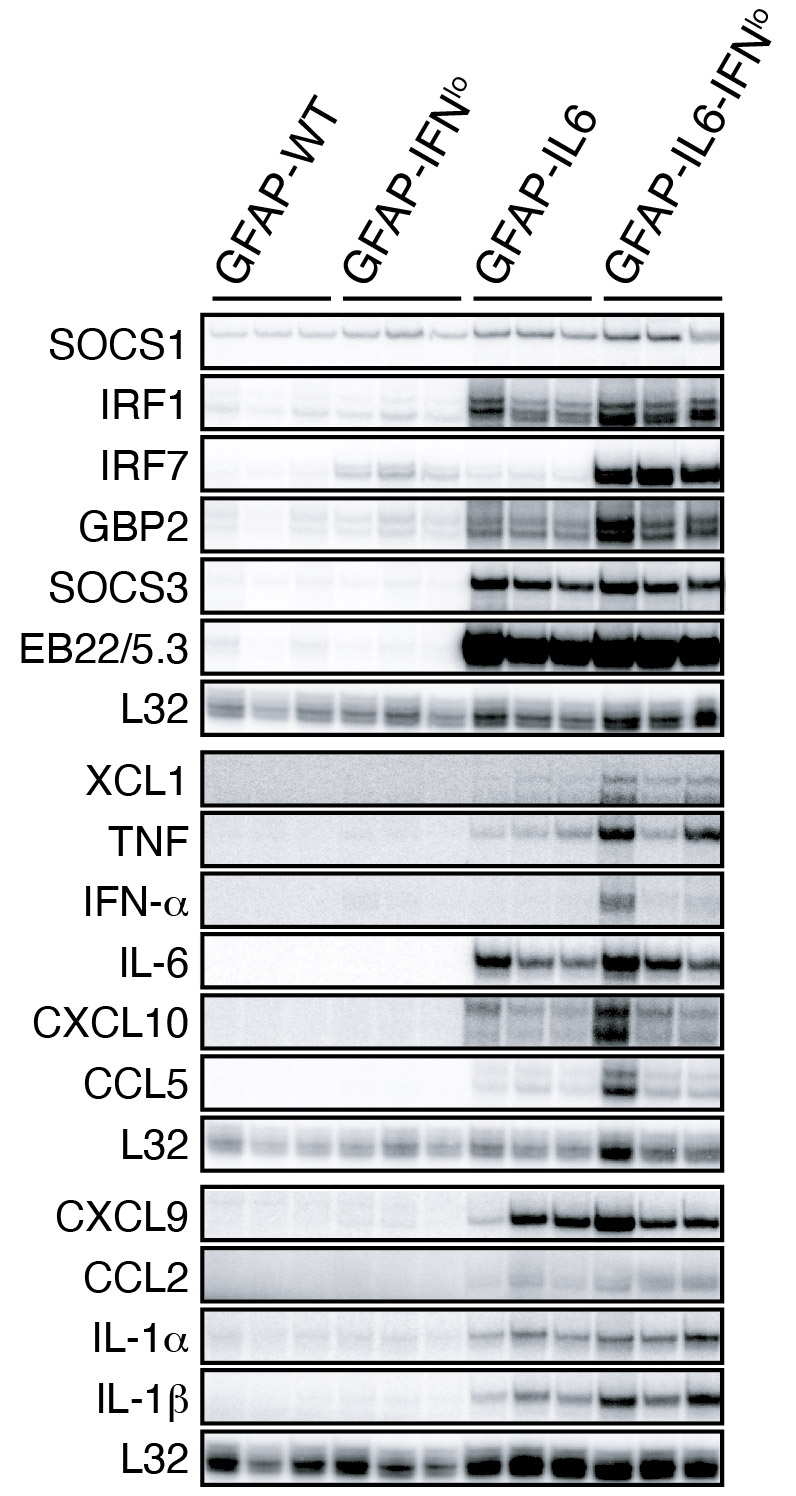

Supplement: Supplementary file 1 [file Image_1.jpeg]

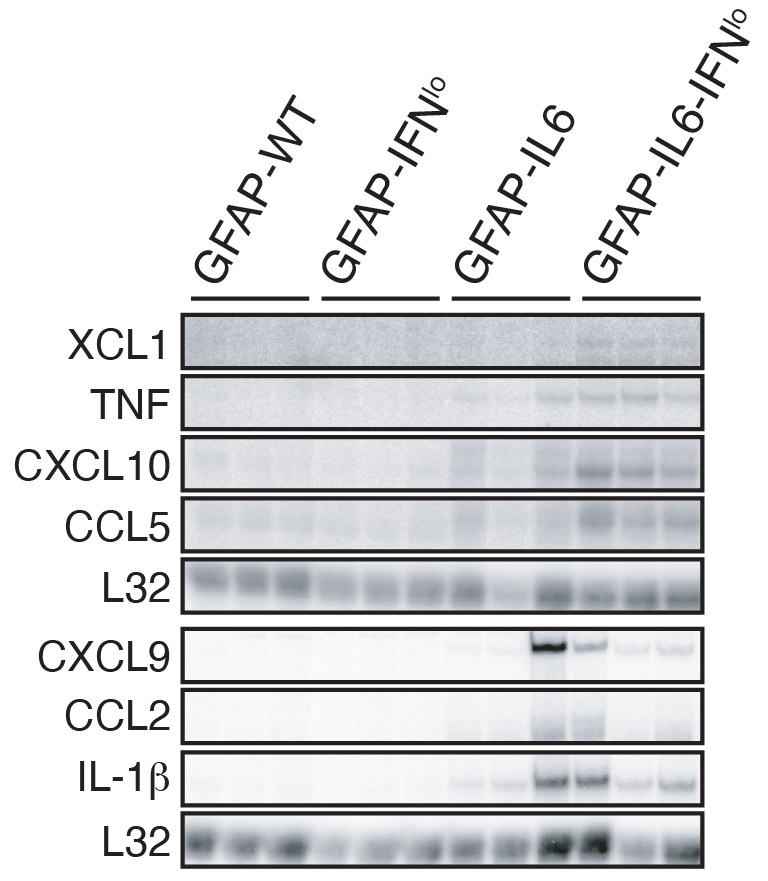

Supplement: Supplementary file 2 [file Image_2.jpeg]
