# Supplementary material for: Interleukin-6-induced neuroinflammation is exacerbated by subclinical levels of interferon-α
Source: Front Neurosci. 2025 Jun 19;19:1586400. doi: 10.3389/fnins.2025.1586400 (PMC12223566; doi:10.3389/fnins.2025.1586400)
Supplement: Supplementary file 5 [file Data_Sheet_1.docx]

Supplementary Material

# Supplementary Data

No supplementary data produced.

# Supplementary Figures and Tables

## Supplementary Figures

**Supplementary Video 1.** **Typical clinical phenotype of GFAP-IL6-IFN^lo^ mice.** Representative video of the phenotype observed, from a 35-week-old GFAP-IL6-IFN^lo^ mice.

**Supplementary Video 2. Balance beam performance of GFAP-IL6-IFN^lo^ mice at 24 weeks of age.** Representative video of the balance beam performance observed in GFAP-IL6-IFN^lo^ mice at 24 weeks of age. Mice were tested for the time taken to traverse the full length of the beam and the number of footslips made over 5 trials. For mice that fell off the beam, trials were repeated until they had successfully completed 5 trials.

**Supplementary Figure 1.** **Expression of selected genes in the cerebellum of mice at 24 weeks of age.**RPA autoradiographs used to quantify prototypic pro-inflammatory gene, IL-6-regulated gene and IFN-regulated gene transcripts in cerebella of GFAP-WT, GFAP-IL6, GFAP-IFN^lo^ and GFAP-IL6-IFN^lo^ mice at 24 weeks of age. Densitometric quantifications of autoradiographs were performed for gene expression analysis, normalized to the L32 loading control.

**Supplementary Figure 2.** **Expression of selected genes in the cerebellum of mice at 8 weeks of age.**RPA autoradiographs used to quantify prototypic pro-inflammatory gene, IL-6-regulated gene and IFN-regulated gene transcripts in cerebella of GFAP-WT, GFAP-IL6, GFAP-IFN^lo^ and GFAP-IL6-IFN^lo^ mice at 8 weeks of age. Densitometric quantifications of autoradiographs were performed for gene expression analysis, normalized to the L32 loading control.
